# Machine Learning and Non-Affective Psychosis: Identification, Differential Diagnosis, and Treatment

**DOI:** 10.1007/s11920-022-01399-0

**Published:** 2022-11-18

**Authors:** Maria Ferrara, Giorgia Franchini, Melissa Funaro, Marcello Cutroni, Beatrice Valier, Tommaso Toffanin, Laura Palagini, Luigi Zerbinati, Federica Folesani, Martino Belvederi Murri, Rosangela Caruso, Luigi Grassi

**Affiliations:** 1 Department of Neuroscience and Rehabilitation, Institute of Psychiatry, University of Ferrara, via Fossato di Mortara 64/A, Ferrara, Italy; 2 Department of Psychiatry, Yale School of Medicine, 34 Park Street, New Haven, CT USA; 3 Department of Physics, Informatics and Mathematics, University of Modena and Reggio Emilia, Via Campi 213/B, Modena, Italy; 4 Department of Mathematics and Computer Science, University of Ferrara, Via Macchiavelli 33, Ferrara, Italy; 5 Harvey Cushing/John Hay Whitney Medical Library, Yale University, 333 Cedar St., New Haven, CT USA

**Keywords:** Machine learning, Psychotic disorders, Schizophrenia, Clinical high risk, Digital phenotyping

## Abstract

**Purpose of Review:**

This review will cover the most relevant findings on the use of machine learning (ML) techniques in the field of non-affective psychosis, by summarizing the studies published in the last three years focusing on illness detection and treatment.

**Recent Findings:**

Multiple ML tools that include mostly supervised approaches such as support vector machine, gradient boosting, and random forest showed promising results by applying these algorithms to various sources of data: socio-demographic information, EEG, language, digital content, blood biomarkers, neuroimaging, and electronic health records. However, the overall performance, in the binary classification case, varied from 0.49, which is to be considered very low (i.e., noise), to over 0.90. These results are fully justified by different factors, some of which may be attributable to the preprocessing of the data, the wide variety of the data, and the a-priori setting of hyperparameters. One of the main limitations of the field is the lack of stratification of results based on biological sex, given that psychosis presents differently in men and women; hence, the necessity to tailor identification tools and data analytic strategies.

**Summary:**

Timely identification and appropriate treatment are key factors in reducing the consequences of psychotic disorders. In recent years, the emergence of new analytical tools based on artificial intelligence such as supervised ML approaches showed promises as a potential breakthrough in this field. However, ML applications in everyday practice are still in its infancy.

## Introduction

Psychosis affects 3% of the global population over their lifetime [1, 2]. The economic burden of these mental disorders is also substantial, both in terms of lost work productivity and direct costs (medications, hospitalizations) [3]. Individuals affected by psychosis have a high likelihood of relapse, with reported rates up to 81.9% five years after an initial recovery [4]; patients also have a higher risk for suicide, especially during the first episodes [5]. Psychosis is still considered a chronic condition associated with considerable disability [2]: in fact, the rates of recovery from psychosis remain low (median is 13.5%) [6] and individuals affected suffer from an astonishing premature mortality [7]. The personal toll suffered by patients and their caregivers in addition to the economic burden for the community make psychosis prevention and early treatment a public health priority [8].

Consistently, with the critical period hypothesis [9, 10], an overwhelming amount of evidence supports the benefits of implementing early detection and intervention in psychotic disorders [11]. Early detected patients show less severe symptoms at care presentation, better global functioning and an overall better quality of life at 2 years [12], lower rates of suicidal behaviors [5] and risk of hospitalizations [4], reduced encounters with the criminal justice system [13, 14], and overall better outcomes up to 10 years after their first episode [15].

Psychotic disorders are usually preceded by a period of variable duration characterized by subthreshold symptoms of psychosis, known as prodromes [16, 17], that include suspiciousness, social withdrawal, diminished attention and concentration, and a drop in overall functioning [16, 17]. However, not all the subjects who will experience prodromal symptoms, or defined as being at high risk for psychosis (CHR-P), will progress to frank psychosis [17–21]. Identifying biomarkers that could be used to determine who will progress to psychosis would allow the delivery of preventive measures to the subgroup that would benefit the most [22].

Studies have struggled so far to identify valid biomarkers of progression to psychosis [23] or schizophrenia [24]. Promising candidate biomarkers have been tested in multiple domains in patients with schizophrenia and individuals with prodromes of psychosis: genetic (e.g., NRG1 gene) [25, 26], brain structures (e.g., gray matter loss, increased levels of neurotransmitters) [27, 28], neurocognitive performance (e.g., working memory, attention deficit) [29], and neuroendocrine anomalies [30].

Within the many strategies that have been tested during the past year to improve the detection and outcomes of psychosis, machine learning (ML) methodologies have seen a tremendous development [31–34]. The term machine learning refers to the subdomain of statistics and computer science focusing on the elaboration of complex algorithms able not only to build models from often huge datasets but also to improve their accuracy, imitating the way humans learn. ML can be employed to uncover patterns of risk of conversion to psychosis that could enable clinicians and stakeholders to implement timely intervention to delay or prevent psychosis [35•]. ML tools offer promise for early detection (i.e., to detect first signs of psychosis and help with differential diagnosis) and prediction of treatment response [33]. Lastly, ML could offer a tool to analyze data obtained from Electronic Health Records (EHRs): this represents a complex but invaluable source of information that is routinely collected in a routine clinical setting but infrequently analyzed because of the poor quality of the data, the lack of standardized instruments, and the large volume of information.

Given the tremendous development of ML approaches to analyze data regarding non-affective psychosis, a review of the most current literature is needed.

## Methods

This narrative review will cover the most relevant findings on the use of ML techniques in the field of non-affective psychosis, by summarizing the studies published in the last 3 years.

The authors searched the following electronic databases: Ovid MEDLINE, Embase, Psycinfo, and Cochrane.

The search used a combination of controlled vocabulary and free-text terms to capture the concepts of “psychosis” and “machine learning.” Example searches are as follows: bipolar or delusion* or hallucination* or psychoses or psychosis or psychotic or schizophren* or schizoaffective or mania or manic and “artificial intelligence” or “deep learning” or “machine learning” or “neural network*.” In addition, a manual search was conducted based on literature references of relevant articles. We focused on recent articles in the English language. Full-text review of the included studies was carried out, and data were extracted on study’s characteristics and outcomes.

## Results

Given the lack of standardized identifiable biomarkers for psychosis, research has explored different sources of reliable information that could be analyzed by means of ML to improve their accuracy and statistical power.

Specifically, sociodemographic information, EEG, neuroimaging (MRI, PET), neurocognitive assessments, psychometric scales, genetic genotyping, blood and CSF immunology, digital phenotyping (e.g., social media, smartphone use), and speech profiling have been investigated.

The studies included in this review employed an assortment of ML techniques, mainly supervised algorithms, comparing their performance in various clinically significant tasks.

The most frequent types of data explored were neuroimaging data, mostly from MRI, followed by EEG, language characteristics, and genetic information. There was considerably less evidence regarding the immediate clinical utility of analyzing digital phenotyping data and electronic health records (EHRs). This difference might be explained by the better suitability of the certain type of information to be processed by currently established ML algorithms, as well as the wide heterogeneity in data sources and structure. The technique that was most frequently applied in the included studies was SVM, while unsupervised ML algorithms were rarely employed, principally for data pre-processing, for example adopting PCA for dimensionality reduction.

The main sources of information that have been employed in ML algorithms will be summarized below.

### Socio-Demographic Factors

Given the complex interconnectivity of this type of data, ML might represent a powerful tool able to untangle it and extract relevant patterns. In our review, the studies addressing this challenge employed socio-demographic data to predict the quality of life, healthcare services utilization, transition to psychosis, treatment response, and functional recovery. The combination of socio-demographic factors with other markers, such as clinical features, appeared to improve the accuracy of the ML algorithms.

Beaudoin et al. analyzed with a LASSO algorithm (a regression analysis method that uses variable selection and regularization to improve the predictability and interpretability of the produced statistical model) a cohort of 919 patients with schizophrenia and found patients and parents’ educational level (most likely also associated with socio-economic status) were strong predictors of quality of life, also, female gender was a strong predictor [36].

In order to build the NAPLS2 calculator [37], Koutsouleris et al. included demographic and clinical features and found that a supervised ML approach was able to have a balanced accuracy of 68% in the identification of those individuals who will subsequently transit to psychosis (specificity was 73%, sensitivity 63%) [38].

In order to predict resources used by patients with non-affective psychosis, logistic regression, classification tree, and random forest methods were applied: Kwakernaak et al. found that the number of psychotic episodes, paid employment, and engagement in social activities affected healthcare consumption [39]. Moreover, the random forest method was found to be the best suited to model risk factors. Legge et al. found that ML approaches such as the conditional inference random forests model identified age at onset, premorbid IQ, and poor social adjustment to be the best predictors of treatment-resistant psychosis, with an accuracy of 0.59, mirroring findings from regression analyses [40].

Similarly, Leighton found that clinical routine data assessment such as employment status at baseline and severity of psychotic symptoms (suspiciousness, hostility, delusions) were able to positively or negatively predict rates of functional recovery respectively (measured as being employed, in education, or training), with an accuracy of 85% by applying elastic net regularized logistic regression models [41].

EEG has often been investigated as a potential tool for early identification: it is a non-invasive assessment, versatile, and with a limited cost [42•]. In our review, the most promising results obtained by exploiting encephalographic data were achieved in discriminating individuals with schizophrenia from healthy subjects and in predicting response to clozapine and ECT [43]. As expected, a generally lower accuracy was noted for the algorithms seeking to classify psychotic and depressed patients.

For instance, Baraditis demonstrated that microstate alterations obtained by resting state EEG and analyzed with SVM supervision were able to discriminate individuals with schizophrenia by healthy controls, with high accuracy (82.7%), sensitivity (83.5%), and specificity (85.3%) [44]. Deep learning convolution neural network was successfully applied to examine multi channels auditory related EEG single trials to distinguish subjects with schizophrenia from healthy controls with an accuracy of 78% [45]. A linear discriminant analysis classifier was successfully applied to test if resting state EEG was not only able to discriminate schizophrenia patients from controls (with an accuracy of 80.66%) but also to stratify patients based on their symptom severity, with an accuracy as high as 88.10% for positive symptoms [46]. Masychev et al. found that patients with schizophrenia have distinctive features at the auditory odd-ball P300 EEG (a measure of brain connectivity, where the presentation of a continuous series of similar tones at a somewhat moderate rate, and the reaction of the participant to this “oddball” stimulus is recorded with EEG) when analyzed by means of supervised ML tools with an accuracy of 92.68% [47]. Moreover, the inferior frontal gyrus EEG features, analyzed with supervised ML methods, most accurately classify schizophrenia patients from controls (accuracy 78.95%) and positive from negative type schizophrenia (accuracy 89.29%) [48]. Linear discriminant analysis and SVM classifiers were also applied to data drawn by the application of EEG to distinguish features of the disease in subjects with schizophrenia from others with depressions, or controls: the SVM classifier showed good accuracy in distinguishing schizophrenia or depressed patients from controls (71.31% and 74.55% respectively), lower performance in distinguishing patients with schizophrenia by those with depression (59.71%) [49]. Finally, Ciprian et al. developed a linear discriminant analysis algorithm to study auditory EEG measures of connectivity activities in the brain of 57 individuals with schizophrenia, to predict response to clozapine treatment with an impressive accuracy of 95.83% [50]. From the same group, Masychev et al. applied a two-step ML analysis, that was able to distinguish most responders to clozapine from least responders, with an accuracy of 89.90% [43]. Moreover, resting-state EEG was found to be a good predictor of response to ECT in patients with schizophrenia [51] (total accuracy 92.68%): transfer entropy, which represents information flow at the EEG, was used as the key information in the random forest method. These studies provided further evidence to support the use of ML tools to analyze EEG data to discriminate the best candidates to clozapine or ECT treatment.

### Language

Given that psychotic disorders are often characterized by disorganized speech (loosening of associations) and content disorders (delusions, echolalia) [52], investigators have applied a variety of natural language (NLP) techniques supervised linear discriminant analysis and supervised leave-one-subject-out cross validation + convex hull classifier to perform speech analysis to identify schizophrenia early in the course of the disease. In the studies included in this review, both form and content features of speech were examined not only in identifying psychosis or transition to illness but also in differentiating schizophrenic patients based on symptom clusters. In our sample, the speech analysis of individuals at clinically high risk for psychosis appears to provide the best results in terms of accuracy.

A study conducted in prodromal individuals found that language alterations including reduced usage of possessive pronouns and semantic coherence had an 83% accuracy in predicting psychosis onset in the training dataset (and 79% in a further independent prodromal sample) and an accuracy of 72% in discriminating patients who recently converted from psychosis from controls [53]. This finding was confirmed by Rezaii et al. that investigated speech in prodromal individuals [54]: progression to psychosis was predicted by low levels of semantic density and an increased tendency to talk about voices and sounds (with an impressive 90% of accuracy, analyzed by a supervised neural network). Speech profile was analyzed with several ML tools, such as supervised gradient boosting [55], latent semantic analysis [56•, 57], random forest [55, 56•], SVM with radial basis function [58•], and multi-layer perceptron [58•]. In their extensive reviews, Ratana et al. [58•], Corcoran et al. [59•], and De Boer et al. [56• ,^60^] provided an overview of the use of speech analysis such as semantic coherence, semantic density, and acoustic analysis via ML, especially within the NLP processing framework, to detect early signs of psychosis: such tools should be considered reliable and valid in the challenging field of early detection of psychosis and major psychiatric disorders. Also, speech analysis could be a useful tool to detect subjects with schizophrenia within the general population, by analyzing their written excerpts transcribed from their verbal utterances, employing supervised techniques (word2vec, SVM with radial basis kernel) [53]. Results from the study by [57] Sarizynksa-Wawer et al. were superior in terms of accuracy than clinically-based assessment, with an accuracy of 80%, highlighting the need to include such analysis in routine evaluation. Within this line of investigation, Tan et al. found that schizophrenia patients had higher incidences of speech aberrance across five types of variables [55]: utterance, single words, speaking rate, turns, and formulation errors. By using supervised gradient boosting and random forest algorithm, 21 speech variables across the above-mentioned types were significant classifiers for a schizophrenia diagnosis with a specificity and sensitivity up to 90% for both models. Moreover, ML techniques can distinguish subjects with positive or negative symptoms based on their speech acoustics, with an accuracy of 86.2%, as shown by De Boer et al. [60].

### Digital Phenotyping

Given the availability of passive data from electronic devices such as smartphones or smartwatches, it is easy to postulate that ML techniques have great potential to become extremely useful for analyzing these large volumes of data for the early identification of psychosis or the transition between the different phases of illness [53, 61–63]. Researchers scanned Facebook content, texts, and Google searches to be able to predict psychosis or relapse applying SVM and random forest that mastered the task with a maximum accuracy of 96%. More so, latent semantic analysis can be extremely helpful to monitor social media in order to screen for and predict transition to psychosis, as summarized in Feldman et al. review [64]. For example, a random forest algorithm was able to distinguish the social media post text of subjects with schizophrenia from those of healthy control with an accuracy of 96% [65]. SVM algorithm and random forest were valid tools to predict a diagnosis of schizophrenia spectrum disorder and a relapse, with an AUC of 0.74 and 0.71 respectively [66] by analyzing text originating from Google searches. Moreover, by analyzing Facebook activity archives with a supervised random forest, the algorithm was able to distinguish subjects with schizophrenia, from those with mood disorders or healthy controls, with high accuracy. More importantly, the analysis of Facebook content alone (e.g., choice of words, punctuation) was able to predict a diagnosis over a year in advance of hospitalization. A validated, structured questionnaire was administered through the smartphone to 260 patients with psychosis and 212 controls, in order to capture mental states daily: within the various ML techniques applied (supervised random forest, SVM, Gaussian processes, logistic regression, and neural networks), the SVM with radial kernel achieved an accuracy of 82% in distinguishing emotional patterns of patients from controls [67].

### Blood and CSF Biomarkers

Blood biomarkers have also been studied as potential candidates that could identify individuals at risk of developing psychosis early in the course of illness [25]. Some of the studies we analyzed focus their attention on plasma proteomic data and inflammatory alteration (such as neurotrophins and oxidative stress markers) in cerebrospinal fluid. These markers combined with ML techniques were able to discern between SCZ and BD and to predict progression to psychotic [21]. Peripheral plasma proteomic data (mostly indicating a dysregulated complement and coagulation cascade) paired with baseline clinical data were successfully used to identify those individuals who will convert from clinical high risk to frank psychosis: ((AUC), 0.95) [68]. Moreover, the model was able to predict who, 6 years later, will have psychotic experience (PPV, 67.8%; and NPV, 75.8%) [68]. Different algorithms have also been tested to analyze CSF alterations to investigate if subjects with schizophrenia show compartment-specific alterations from classical inflammatory CNS disease with high accuracy (0.88 psychosis vs intracranial hypertension) [69]. Supervised ML has been also used to analyze biomarkers including neurotrophins, inflammatory (IL-10), and oxidative stress markers (e.g., glutathione peroxidase), associated with psychosis: the supervised algorithm failed to distinguish BD from SCZ (accuracy = 49%), but was able to reach a prediction accuracy of 77.5% and 72.5% to identify, respectively, patients with SCZ and BD from controls [70]. It is possible that affective and non-affective psychosis share the same pathophysiological mechanisms so that it is hard to distinguish them by using these biological markers [71, 72].

### Genetics

The high degree of complexity in genetic data represents a hurdle for traditional statistical genetics; however, with the exponential growth of computing power and datasets volume of recent years, ML has emerged as an attractive option. As expected, the studies in our review showed a wide range of performance rating, likely caused by high heterogeneity in study design. Even for this type of biomarker, the integration with different data sources, e.g., brain morphology or cognitive features, seems to yield better predicting results.

Genetic analysis is also becoming popular in the field, given the initial evidence of a potential role of both inherited as well as de novo mutation variants [105] in neuronally expressed genes [73, 74], contributing to synaptic dysfunction in the pathogenesis of SCZ. Trakadis applied the supervised Extreme Gradient Boosting (XGBoost) with regularization in a case–control study (2545 SCZ and 2545 controls) to identify genetic markers of a risk for developing SCZ (accuracy = 0.85, sensitivity = 0.85, specificity = 0.86) including genes that regulate neurogenesis and neuronal development, synaptic plasticity, memory, and axonal development [75]. A larger schizophrenia case–control study of 11,853 subjects applied supervised support vector machines (SVM) with linear and radial basis function kernel methods to identify possible genes contributing to the risk of developing SCZ [76]: the sensitivity and specificity were lower compared to the Trakadis study (AUC 0.60–0.66); moreover, its prediction accuracy was lower than that obtained by the use of polygenic risk score [77, 78]. A further study [79] tried to test the effect of 77 risk loci known to be strongly associated with SCZ to predict six different cognitive phenotypes in subjects with schizophrenia, finding that polygenic risk scores and random forest had similar predictive strength and error. However, as highlighted in the systematic review conducted by Bracher-Smith et al. [31], ML methods performance measures have a wide range of abilities (from 0.48 to AUC 0.95) and are still inadequate for prediction modeling: the most commonly employed were supervised naïve Bayes, k-nearest neighbors, penalized regression, random forest, Gaussian processed, SVM, and neural networks. Finally, as Yang et al. underlined in their research on individuals with schizophrenia, ML tools can become even more precise predictive tools when combining genetic and brain morphology data [80]. A further example in this sense is the work by Chen et al. [81, 82] that applied an updated biologically imported machine learning (BioMM) approaches to identify a blood DNA methylation signature that could differentiate schizophrenia from healthy control, and investigate the association of peripheral biomarkers, and neural functioning. Blood-based immunological biomarkers were combined with cognitive data in a multi-domain data integration machine learning model in order to differentiate subjects with schizophrenia from healthy controls with a sensitivity of 84% and specificity of 81% [83].

### Neuroimaging

Given the high clinical heterogeneity of psychiatric disorders, the kaleidoscopic presentation at onset and the dynamic evolution, ML methods have been largely employed to analyze neuroimaging data, alone or in combination with other evaluations including blood biomarkers, neuropsychological tests, and clinical structured assessment. With a very few exceptions represented by the application of unsupervised algorithms (non-negative matrix factorization) to analyze data from magnetoencephalography and structural MRI in first-episode schizophrenia patients [84], most studies applied supervised ML techniques.

The vast majority of the studies focusing on the prediction of psychosis conversion applied ML to resting state MRI. Koutsouleris et al. found that it was possible to increase by 40% the diagnostic certainty of subjects who will convert to frank psychosis by applying the MRI-based biomarker, specifically the prefrontal perisylvian and subcortical brain structures [85, 86]. Transition outcomes were correctly predicted in 80% of test cases using MRI-based predictors, which increased prognostic certainty by 36% (sensitivity: 76%, specificity: 85%) [87].

Kambeitz et al. tried to test an ML tool to predict global functioning outcomes at the individual level, by focusing on cortical area reductions in superior temporal, inferior frontal, and inferior parietal areas, with an accuracy of 82%, underlining the utility of ML in stratifying the risk to progression towards psychosis in ultra-high risk individuals [88, 89].

Resting state MRI alone can help build a machine-learned classifier for diagnosing schizophrenia with an accuracy of 87% [90]. Structural MRI is gaining importance to help differentiate between SCZ and healthy controls, as summarized in de Filippis et al. review [91•], as SVM could reach an accuracy of 100% if combined with more recent ML tools [92]. A parameter that has been studied as a potential biomarker of psychosis is the disrupted functional asymmetry: this value in the left thalamus discriminated control vs FEP/UHR individuals with high sensitivity (68.42% and 81.08% respectively) [93]. Antonucci et al. found that SVM built with a repeated nested cross-validation framework was able to distinguish schizophrenia patients from HC by computing attentional control task in fMRI, to identify a pattern of connectivity alterations, with an accuracy of 66.9% [94]. An explainable deep neural network framework provided insight on some brain-based imaging markers, especially decreased density in the insula and frontal and superior temporal lobe, and reduce white matter in the cingulum, hippocampus, with high accuracy (up to 84%) for gray matter [95]. ML was also employed to discriminate subjects with schizophrenia from healthy control with an accuracy of 0.72% [96], or predict the response to treatment in first-episode drug naïve subjects, with an accuracy of 82.5% [97]. Also, deep neural network models were applied to identify brain abnormalities with an accuracy of 81.5% [98] supervised SVM-RFE combining functional and structural MRI was able to distinguish schizophrenia patients from HC with an accuracy of up to 80% [99], and combining polygenic risk score and structural imaging methods with 71.6% accuracy by exploring data of more than 1000 subjects from eight independent sites across China [100].

Individual structural and functional connectivity networks can also help to distinguish subjects with SCZ from healthy controls, as shown by Arbabshirani et al. [101] and Han et al. [102]. These networks were analyzed by Han et al. using SVM in SCZ and MDD patients. SCNMF (supervised convex nonnegative matrix factorization) was successfully employed to draw the distinct characteristics of the two diagnoses, with an 82.6% accuracy. The inferior parietal lobule, middle cingulate, and cingulate cortex were the most discriminative areas in terms of functional properties: as Zeng et al. pointed out, those regions are the ones involved in the salience, control, and default network [103]. Qureshi et al. focused on functional connectivity as a potential biomarker of SCZ, with an accuracy of 0.99 [104]. Moreover, aberrant connectivity in temporal and occipital regions resulted in a good prediction marker, according to Li et al. [105]. Anticorrelated networks between sub-cortical and cortical areas were found to be a strong marker of schizophrenia (accuracy = 0.69) [106].

Sensorimotor circuits also might play a relevant role in the pathogenesis or clinical presentation of SCZ, as demonstrated by the high accuracy (0.95) of the ML algorithm (SVM) applied by Guo et al. [107]. Linear SVM and nonlinear (decision tree) ML algorithms can be helpful to predict the outcomes of at 1 year, specifically by looking at the dynamics of resting state functional connectivity with the default mode network with an accuracy ranging between 75 and 90% [108]. Finally, an ML algorithm could possibly inform about the risk of developing psychosis in non-affected siblings of patients with schizophrenia, as hypothesized by Morgan et al. based on the results of their study on functional connectivity patterns [109].

Given that MRI scans are easily administered, they should be included in the routine baseline assessment of individuals at their first episode of psychosis, or under evaluation for psychosis risk. The advantages are considerable: first, MRI can pick up rare organic causes of psychosis (e.g., tumors), but they can also track the progression of psychosis in the brain (e.g., measures of cortical thickness). However, despite being MRI a very promising tool, it has not been fully implemented in clinical practice because of several translational challenges: first, MRI are quite expensive and given the resources constraints in many healthcare services it might be not easy to charge for this “unnecessary cost”, second, neuroimaging sessions take time to be performed (at least 30–40 minutes per session) and again it might not be suitable for working patients (who might have limited time available), or for individuals who struggle with claustrophobia or other forms of anxiety.

### Electronic Health Records

In order to offer a precision medicine approach, it might be necessary to create more population-based data reflecting the real incidence of mental disorders and to use electronic health records (EHRs) [110]. In that regard, EHRs have a lot of potential for speeding up clinical research and for helping clinicians to predict the outcome for each patient [111]. For example, Holderness et al. used two types of artificial neural networks (ANN), multiple multilayer perceptron (MMP) and RBF, to predict whether sentences in a patient’s EHR are linked to one or more of the identified risk factor domains for readmission [112]. Overall agreement was good when compared to annotators, with a mean accuracy of 80.5%. Senior et al. [113] developed a natural language processing technique in order to extract variables from clinical notes to predict risk factors for suicide in 57 patients with SCZ and BD. In comparison to the manual evaluation, the overall accuracy was good (the overall micro precision was 0.77, recall was 0.90, and F1 was 0.83.).

### ML to Predict Treatment Outcome

Multiple attempts have been made to predict the course of psychosis in research using ML [114, 115] with inconsistent accuracy (up to 70%). Researchers tried to identify some markers to forecast that, for example, depressive symptoms, poorer educational attainment, functional problems, unemployment, and unsatisfied psychosocial demands and antipsychotic medication were some of the strongest ones. Lower education, functional deficits, unemployment, and unmet psychosocial needs discovering a marker to foretell the response to antipsychotic is a promising way to apply the ML technique: resting-state functional MRI combined with LASSO was used to evaluate the clozapine response in 3 months.

One of the first studies that used ML to predict outcomes in an FEP sample was conducted by Koutsouleris et al.: lower education, functional deficits, unemployment, and unmet psychosocial needs were identified as the most accurate predictors of 4- and 52-week outcomes (with an accuracy of 73.8–75%) [38, 87]. A similar study was conducted a few years later on a sample of 523 subjects diagnosed with schizophrenic disorders, by applying a linear SVM and recursive feature elimination within a nested cross-validation design to recognize patterns in a wide range of genetic, clinical, and environmental variables. The accuracy in predicting symptomatic outcome was 62.2 to 64.7%. The most important predictors were global assessment of functioning areas, psychotic and depressive symptoms, broad quality-of-life indicators and overall functioning, and antipsychotics use; psychosocial needs were also confirmed as a strong predictor [116].

Antipsychotics represent the core component of any comprehensive psycho-social treatment for psychosis [117]. However, two features have characterized psychosis treatment so far: no biomarker or clinical characteristic has been identified as an indicator of antipsychotic response [118]; second, treatment-resistant psychosis emerges in up to a third of patients [119, 120] and there are no reliable tools to predict the lack of response to a certain antipsychotic. Thus, ML holds the promise to help clinicians in moving towards a more personalized pharmacotherapy to increase the accuracy and relevance of predictions for pharmacological treatment outcomes [121]. Sarpal et al., using resting-state functional MRI, found that 91 regions that have functional connections with the striatum were an accurate prognostic tool for treatment response to antipsychotic in acutely psychotic patients [122]; however, the accuracy was 78%. A LASSO algorithm was also used to predict clozapine response at 3 months [123].

A brain source localization (BSL) procedure using the linearly constrained minimum variance (LCMV) beamforming approach was also explored: response to clozapine treatment can be predicted by the symbolic transfer entropy features with an accuracy of 95.83% [124].

Given the high incidence of treatment-resistant schizophrenia, a growing number of studies are delving into the pharmacogenomics of antipsychotics, but despite the efforts that have been made so far, no reliable predictive markers have been identified that might be employed in clinical management and that could enhance the quality of life of these patients. However, recent studies implemented ML methods to investigate the genetics of treatment-resistant schizophrenia, reporting promising findings that could pave the way for the application of pharmacogenomics in the clinical practice [125].

## Discussion

This review summarizes the findings of the most current literature on the application of ML techniques for the identification, diagnosis, and treatment of non-affective psychosis.

Most of the studies focused on neuroimaging data, especially MRI, with a specific focus on cortical area reductions in superior temporal, inferior frontal, and inferior parietal areas that could help identifying the progression to psychosis in UHR individuals. The disrupted thalamus functional asymmetry in fMRI matched with SVM and deep neuronal model showed to be a useful marker to distinguish SCZ from healthy volunteers as well as the decreased density in the insula, frontal and superior temporal lobe, or the reduced white matter in the cingulum and hippocampus, or connectivity alterations (tested with a computing attentional control task in fMRI). ML techniques can also be a helpful tool to predict treatment outcome: for example, 91 regions that have functional connections with the striatum have been studied with the LASSO algorithm. The linearly constrained minimum variance (LCMV) was also utilized to predict clozapine response after 3 months.

When dealing with socio-demographic data, the best ML method was found to be the random forest that was able to identify the age at onset, premorbid QI, and poor social activities as the most frequent predictor of psychosis. A lot of studies focused on resting state EEG: SVM seems to be one of the most accurate methods to discriminate healthy controls from patients with SCZ or depression. Moreover, the supervised ML technique matched with auditory EEG revealed to be a useful tool to predict the response to clozapine and ECT treatments.

Supervised ML techniques such as SVM and gradient boosting were used to test language features to distinguish psychotic from healthy people. Low semantic density, higher prevalence of speech aberrance, and increased predisposition to talk about voices and sounds seem to be strictly associated with SCZ patients. Furthermore, random forest and SVM were shown to predict psychosis and relapse by monitoring Google searches and social media posts.

Proteomic and inflammation alterations (such as neurotrophins, IL-10, and glutathione peroxidase) combined with ML technologies were able to distinguish healthy controls from patients with bipolar disorders or schizophrenia. Even genetic features could be some interesting options to explore because it is known that neuronally expressed genes are involved in the etiology of SCZ by causing synaptic disruption. Both SVM and other ML were tested but it was clear that these information about gene loci and DNA methylation need to be combined with cognitive or neuroimaging data to be more accurate.

Such findings are very promising, especially when ML is employed to decipher possible predictors of psychosis by using neuroimaging data; however, it has not been fully implemented in clinical practice because of several translational challenges (cost, resources restraints, claustrophobia). Moreover, almost none of the study provided results stratified by biological sex: given that psychosis has a different epidemiology, clinical presentation, and pathophysiology in the two sexes [126–128], there is the necessity to develop adequate data analytic strategies to account for this important biological characteristic.

From a strictly technical standpoint, almost all the studies considered in this review applied supervised ML techniques, which is quite common in the context of both classifications (binary and multiple) and regression. On the other hand, studies incorporating unsupervised ML techniques to preprocess the data, for example operating a dimensionality reduction or identifying the most relevant variables without the need for human intervention, obtained better performance measures. Most of the studies used SVMs, supervised classifiers operating by finding the optimal decision boundary to distinguish two classes in a dataset, which can be either linear or non-linear. The successful use of SVM is likely driven by the great confidence gained by the field in using this technique as well as the wide availability of fully automatic computer tools for its use. However, it is interesting to note that overall performance (in the case of binary classification) varied from 0.49, which is to be considered very low (i.e., noise), to over 0.90. These results are fully justified by different factors, some of which may be attributable to the preprocessing of the data, the wide variety of the data, and the a-priori setting of hyperparameters (parameters not learned during the training phase of the methodology but set by the architect of the software 10.3934/mine.2023012).

## Conclusions

As a discipline, psychiatry suffers from some main challenges: first, there is a lack of biological markers of disease and prognosis, second, there is a large heterogeneity in clinical presentation, treatment response, and progression, and third, most of research studies, with a very selected and neat population, do not always translate into real-world practice. Thus, even ML shows a limited accuracy despite having a great potential. While DL techniques would not be able to provide any insights into the psychopathology of psychosis, they might unveil a pattern, by using large and various sources of data (biological, clinical, passive data from wearable electronic devices on patients, EHRs) not based on epistemological assumptions. This will allow a better understanding of the disease and provide useful tools to personalize both identification and treatment.

## Abbreviations

AI: Artificial intelligence
ANN: Artificial neural networks
AUC: Area under curve
BD: Bipolar disorder
CHR-P: Clinical high risk for psychosis
DL: Deep learning
EEG: Electroencephalography
EHRs: Electronic health records
FEP: First episode psychosis
ML: Machine learning
MMP: Multiple multilayer perceptron
MRI: Magnetic resonance imaging
NPV: Negative predictive value
PCA: Principal component analysis
PPV: Positive predictive value
RBF: Radial basis function
RF: Random forest
SCZ: Schizophrenia
SVM: Support vector machine
